# A Case of Initial Missed Diagnosis of Fatal Posterior Atlantoaxial Dislocation Without Odontoid Fracture

**DOI:** 10.1002/ccr3.71173

**Published:** 2025-11-06

**Authors:** Tetsuhiro Hagino, Tetsuo Hagino, Tetsuro Ohba

**Affiliations:** ^1^ Department of Orthopaedic Surgery, Faculty of Medicine University of Yamanashi Yamanashi Japan; ^2^ Department of Orthopaedic Surgery National Hospital Organization Kofu National Hospital Kofu Yamanashi Japan

**Keywords:** cardiopulmonary arrest, odontoid fracture, posterior atlantoaxial dislocation, upper cervical spine injuries

## Abstract

We report a rare case of fatal posterior atlantoaxial dislocation without odontoid fracture. Attention should be paid to the possibility of delayed or missed diagnosis of cervical spine injury in patients with disturbance of consciousness.

## 1 Introduction

Posterior atlantoaxial dislocation (PAD) without odontoid fracture is an exceptionally rare and severe injury. Only a few dozen cases have been reported in the literature [1, 2, 3, 4, 5], and prior reviews have noted just ten survivors of this injury [1, 2, 3, 4, 5]. These dislocations typically result from high‐velocity hyperextension trauma that causes severe spinal cord distraction, frequently resulting in immediate death [6]. Indeed, many cases may only be detected at autopsy, suggesting that this condition is underrecognized [7]. Survivors, when reported, often present with surprisingly mild or no neurological deficits despite the dramatic dislocation [1, 2, 3, 4, 5], making early recognition challenging.

This case is particularly informative because it demonstrates a PAD without fracture that was initially missed on routine imaging and proved fatal despite prompt resuscitation efforts. The combination of an uncommon injury pattern, an initially missed diagnosis, and a fatal outcome underscores the importance of systematic cervical spine CT with multi‐planar reconstruction in trauma patients with altered consciousness.

Upper cervical spine injuries occur frequently, accounting for approximately 20% of acute cervical spine injuries [6], and may cause permanent disability or death. We report a rare case of fatal PAD without fracture of the odontoid process associated with head trauma.

## 2 Case History/Examination

He was found in cardiopulmonary arrest at the scene.

**FIGURE 1:**
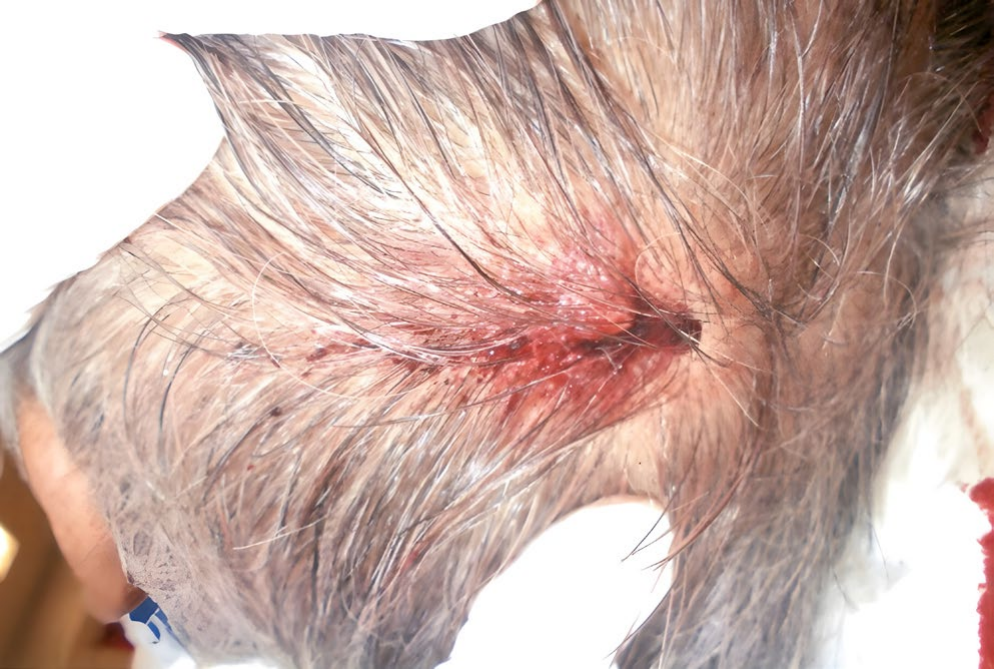
A laceration measuring 4 cm is observed in the left parietal region.

At our hospital, the patient's Glasgow Coma Score was 3 (E1 V1 M1) and Japan Coma Scale score was 300. He was still in cardiopulmonary arrest, and cardiopulmonary resuscitation was continued. A laceration measuring 4 cm was noted in the left parietal region (Figure 1), but no trauma was evident in his chest, abdomen, pelvis, and extremities. Hematology and biochemistry tests conducted immediately after arriving at our hospital showed a high creatinine kinase level of 1196 U/L, suggesting high‐energy trauma, but there was no evidence of anemia or inflammation. Although airway management and chest compression were continued and adrenaline was administered, the patient showed no response and he was pronounced dead 1 h and 30 min after the injury. The type of cardiac arrest rhythm (e.g., pulseless electrical activity or ventricular fibrillation) could not be confirmed (E: Eye response 1, V: Verbal response 1, M: Motor response 1).

## 3 Methods

**FIGURE 2:**
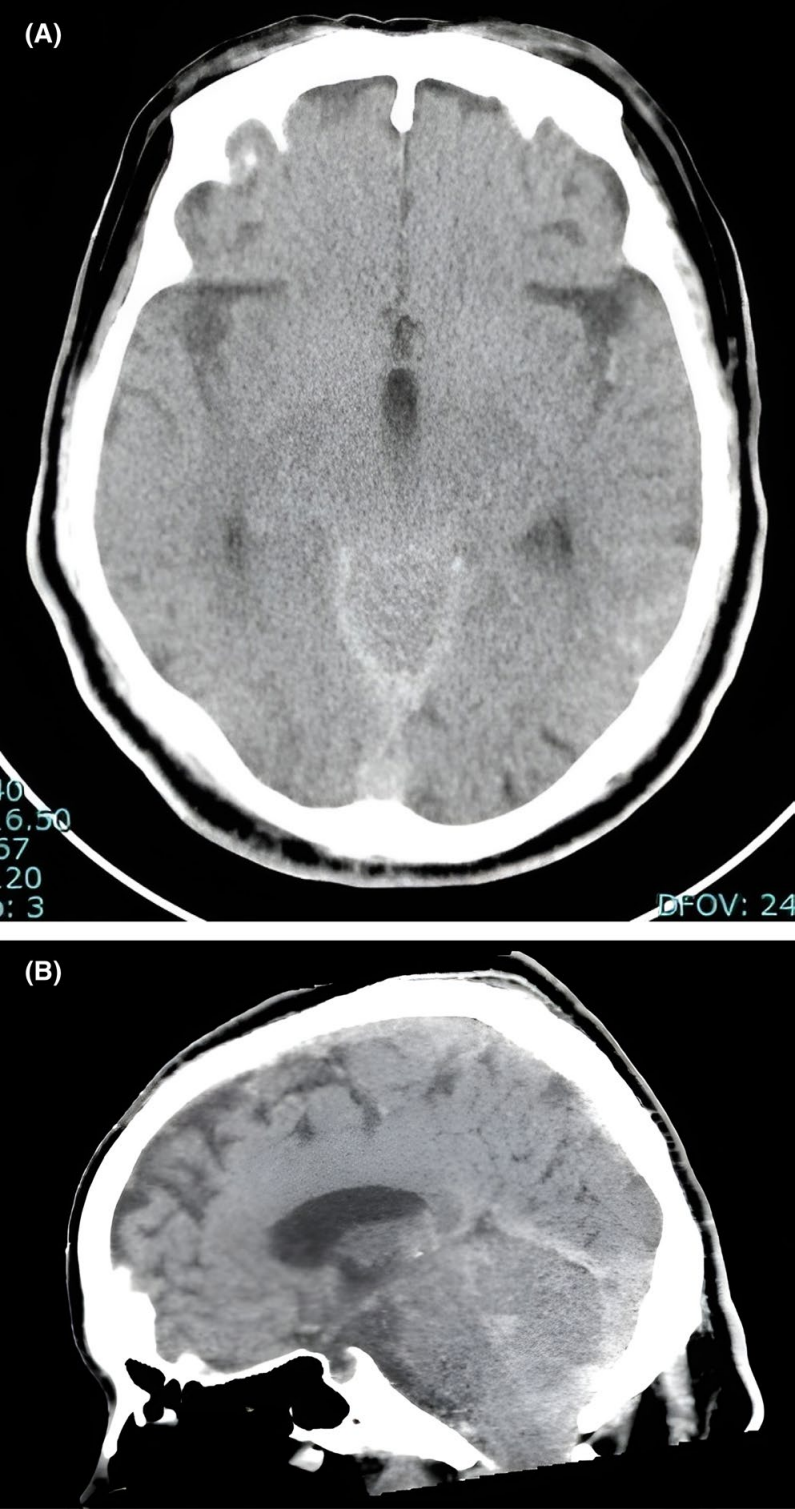
Head CT findings from postmortem imaging. (A) Axial image showing mild subarachnoid hemorrhage and intraventricular hemorrhage. The red arrow indicates mild subarachnoid and intraventricular hemorrhage on axial imaging. (B) Sagittal image showing no evidence of cerebral herniation or brain injury that could have caused cardiopulmonary arrest. The red arrow indicates mild subarachnoid and intraventricular hemorrhage on sagittal imaging.

**FIGURE 3:**
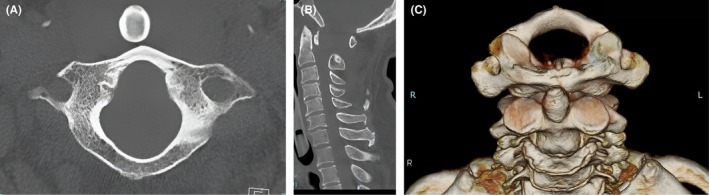
Upper cervical spine CT findings. (A) Axial image showing complete posterior dislocation of the anterior arch of the atlas behind the odontoid process, without definitive fracture. (B) Sagittal image clearly confirming the positional relationship: The anterior arch of the atlas lies entirely posterior to the odontoid process. The red arrow on the 3D volume‐rendered CT image marks dislocation at the same location. (C) Three‐dimensional reconstructed image providing a comprehensive spatial visualization of the posterior dislocation of the atlas relative to the axis.

Imaging was performed during autopsy. Computed tomography (CT) of the head showed intracranial findings of low‐grade subarachnoid hemorrhage and intraventricular hematoma. The emergency physician initially considered head trauma to be the cause of death (Figure 2). At a conference held several hours later, the neurosurgeon disagreed with head trauma as the cause of death because there was no cerebral herniation or significant brain injury that would have led to cardiopulmonary arrest and death. Hence, the CT images were reviewed. On an upper cervical spine CT scan, the odontoid process was noted to be displaced anteriorly, in front of the arch of the atlas. PAD was observed although there was no definitive fracture (Figure 3).

## 4 Conclusion and Results

Based on the above findings, the direct cause of death was determined to be spinal cord injury due to PAD.

## 5 Discussion

Atlantoaxial dislocation is usually caused by hyperextension trauma and is accompanied by odontoid fracture in most cases, frequently leading to immediate death. PAD without odontoid fracture is extremely rare, and only case reports have been published [1, 2, 3, 4, 5]. In the majority of the cases, fatal spinal cord injury is sustained after high‐velocity trauma and is often diagnosed at autopsy [6]. Although autopsy imaging confirmed the cause of death to be spinal cord injury due to PAD in the present case, the cause of death in such cases could easily be missed at postmortem examination; hence the incidence may be much higher than is recognized [1]. In this case, the findings of disturbance of unresponsiveness and cardiopulmonary arrest upon arrival at our hospital led physicians to initially consider that the cause of death was head injury. It should be noted that a diagnosis of cervical spine injury may be delayed as attention is diverted to other serious concurrent injuries including head trauma. In the study of Kitamura et al. [8], among 1313 patients with blunt trauma, 5 patients had severe disturbance of consciousness accompanied by cervical spinal cord injury. Of these 5 patients, 3 developed both respiratory muscle paralysis and quadriplegia, and the levels of injury were C1/2 and C2/3. They concluded that it is extremely difficult to predict the onset of paralysis in patients with severe disturbance of consciousness secondary to head trauma, and when a trauma patient requires a CT scan to detect injuries in other parts of the body, it is important to actively conduct a cervical spine CT scan along with multi‐planar reconstruction. Furthermore, Iwase et al. [7] reported that cervical spine injury may occasionally show CT findings similar to subarachnoid hemorrhage caused by rupture of cerebral artery, indicating that cervical spine injury could be misdiagnosed as a brain disease by postmortem CT. Therefore, caution should be exercised when using postmortem CT for screening trauma.

The atlantoaxial spine is formed by the odontoid process at the center together with the anterior arch of the atlas on the anterior side and the transverse ligament on the posterior side. Due to the weakness of the transverse ligament located posterior to the odontoid process, the majority of atlantoaxial dislocations are due to anterior dislocations. On the other hand, posterior dislocation is a rare condition that occurs concurrently with odontoid or other fractures, or requires total rupture of the atlantoaxial ligaments with displacement of the anterior arch of the atlas beyond the tip of the odontoid process [9]. Haralson et al. [10] reported that the mechanism of posterior dislocation of the atlas without fracture of the odontoid process is hyperextension associated with neck extension caused by a strike to the face or posterior torso. In addition, Xu et al. [6] also reported frequent concurrent occurrence of facial or neck injuries. In the current case, a two‐stage mechanism may be involved; collision from the rear caused hyperextension injury of the cervical spine, preceded or followed by head trauma including laceration of the head and traumatic subarachnoid hemorrhage. However, the exact mechanism remains unclear.

## Author Contributions

**Tetsuhiro Hagino:** conceptualization, investigation, methodology, project administration, writing – original draft, writing – review and editing. **Tetsuo Hagino:** project administration, supervision. **Tetsuro Ohba:** supervision, writing – review and editing.

## Ethics Statement

The authors have nothing to report.

## Consent

Written informed consent was obtained from the patient's family (the patient was deceased) to publish this report in accordance with the journal's patient consent policy.

## Conflicts of Interest

The authors declare no conflicts of interest.

## Data Availability

The data presented in this study is available upon request from the corresponding author. The data are not publicly available due to containing information that could compromise the privacy of the research participants.
